# Upscaling Statistical Patterns from Reduced Storage in Social and Life Science Big Datasets

**DOI:** 10.3390/e22101084

**Published:** 2020-09-26

**Authors:** Stefano Garlaschi, Anna Fochesato, Anna Tovo

**Affiliations:** 1 Dipartimento di Fisica e Astronomia “Galileo Galilei”, Università degli studi di Padova, Via Marzolo 8, 35131 Padova, Italy; anna.tovo@unipd.it; 2 Fondazione The Microsoft Research—University of Trento, Centre for Computational and Systems Biology (COSBI), Piazza Manifattura 1, 38068 Rovereto, Italy; anna.fochesato@unitn.it; 3 Dipartimento di Matematica, Università degli studi di Trento, Via Sommarive 14, 38123 Povo, Italy; 4 Dipartimento di Matematica “Tullio Levi-Civita”, Università degli studi di Padova, Via Trieste 63, 35121 Padova, Italy

**Keywords:** upscaling life science data, statistical patterns inference, big data storage reduction

## Abstract

Recent technological and computational advances have enabled the collection of data at an unprecedented rate. On the one hand, the large amount of data suddenly available has opened up new opportunities for new data-driven research but, on the other hand, it has brought into light new obstacles and challenges related to storage and analysis limits. Here, we strengthen an upscaling approach borrowed from theoretical ecology that allows us to infer with small errors relevant patterns of a dataset in its entirety, although only a limited fraction of it has been analysed. In particular we show that, after reducing the input amount of information on the system under study, by applying our framework it is still possible to recover two statistical patterns of interest of the entire dataset. Tested against big ecological, human activity and genomics data, our framework was successful in the reconstruction of global statistics related to both the number of types and their abundances while starting from limited presence/absence information on small random samples of the datasets. These results pave the way for future applications of our procedure in different life science contexts, from social activities to natural ecosystems.

## 1. Introduction

In the last few centuries, the proceeding of human knowledge in natural and life sciences has been affected by the limited possibilities in collecting data and making them available for the entire research community. Thanks to technological developments in the modern era, science is facing opposite issues. Nowadays, it is possible to collect large amounts of data at a very high speed and lower costs [1,2,3]. Hence, the problem has shifted to the storage of an extensive volume of information into datasets with feasible dimensions for fruitful analysis [4,5]. Moreover, in some cases, this abundance has paradoxically slowed down scientific research, since it is becoming harder to analyse such a huge quantity of data with standard techniques and to extract meaningful information from them [1,2,4,6,7].

This is particularly true in the novel research field of genomics—at the dawn of these studies, the collection of data from DNA sequencing was indeed a limiting factor for making new discoveries and observations. In contrast, thanks to next-generation technologies, today the main obstacle is the storage of new fast-coming data that require massive technological and computational resources to be processed and analysed [5,8,9,10,11,12,13].

The same problem also affects the storage of data generated by the everyday-life human interactions into analysable datasets. Indeed, we can think to social networks like Twitter or e-mail/phone service providers in which every minute new tweets, posts or messages come to circulate within the network thanks to the many users’ activities [14,15,16]. These are examples of systems that generate *big data*, a term used to refer to any large volume of complex data collected at a high rate [17].

This new feature of the contemporary data-driven research has given a boost to the creation of an interdisciplinary vision of science [12,18]. Indeed it is very common to find large collaborations made up by scientists coming from different fields who bring their expertise to tackle the big data problems.

In fact the analysis of such big datasets, often referred as *big data mining*, represents one of the most challenging problem of modern research [4,7,19]. Indeed, processing the entire data overflow coming from either human activities or life science has become an hard task that requires huge computational efforts. To overcome this issue, it would be helpful to be able to properly reduce the amount of collected information in order to end up with data sets that, on the one hand, have manageable dimensions for their analysis, but, on the other hand, allow us to retrieve the important statistical properties of the entire original dataset.

Here, adopting an interdisciplinar mindset, we borrow the statistical framework of upscaling, originally developed for ecological purposes, to deal with big data in an efficient way. Indeed, one common problem in ecology is the quantification of the biodiversity of a given environment. However, surveying the ecosystem in its entirety in order to observe all the different species present in it would require endless resources. Upscaling techniques solve this problem by using suitable statistical models to provide estimates of the biodiversity based on information registered only within a small portion of the ecosystem. In particular, this usually translates into biodiversity estimators used to predict the total number of different species together with their abundances from local knowledge [20,21,22,23,24,25,26]. Hence, by exploiting upscaling approaches, instead of creating a large dataset collecting all the information about an ecosystem in its entirety and extracting the quantities of interest from it, we can limit ourselves to study a sub-sample of the system and still be able to get reliable estimates of some statistical properties of the entire environment, with the advantage of keeping the data acquisition procedure feasible.

The idea of starting from a restricted amount of data to retrieve information about the entire dataset may address a possible way to deal with the storage problem in the big data context—instead of recording the whole data stream produced by a particular system, be it social or natural, it may suffice to only store a fraction of it to unveil and explore some of its main properties. To achieve such a goal, in the Materials and Methods section, we derive within a general setting a novel upscaling estimator from a maximum likelihood approach, independent of the type of data under consideration. Regarding the latter, we built a procedure that, starting from presence/absence information obtained from small random sub-samples of a datastream, allows us to retrieve important statistical patterns, leading to a satisfying overall quantitative description of the entire system. We thus apply our novel procedure to some real-world datasets. In particular, in this paper we consider three different kinds of systems: forest ecosystems, human activities and genomic polymorphisms. More precisely, we analysed data related to tree species observed in a 50 ha area from two tropical rainforests located in Barro Colorado Island and in the Pasoh reserve, respectively [26]. As for human activities, we used four datasets comprising the collection of emails sent from a university department in a two-year period [27] and a selection of posts from the Twitter network, of articles from Wikipedia and of books from the Gutenberg project [28]. Finally, we studied one database on human single nucleotide polymorphisms detected along the *Y* chromosomes of different sequenced male individuals [29,30,31] (see also Supplementary Materials for the availability of all the datasets).

As we will discuss later, with respect to other recently developed upscaling estimators, the novelty of the proposed procedure consists in the difference of the inputs required by the methods. In particular, given the same portion of stored data, we need to use a smaller amount of information. This aspect will translate in a more agile implementation of the framework.

We remark that other procedures exist in literature to deal with the storage problem with big data, such as data compression techniques [32]. These usually stores all the datastream but reduce the bits needed to encode and register the information. The implementation of this kind of algorithms requires advanced computational knowledge and large technological efforts. In contrast, our starting point is the reduction in the data storage requirements. Indeed, we propose an upscaling procedure that, starting from local binary information on the presence/absence of types in small random samples of the whole incoming data allows us to reconstruct basic statistical properties of the entire dataset. This step of storing only partial information of a dataset usually leads to a loss of quality in the predicted quantities. Nevertheless, since the framework is based on basic statistical notions, the advantage in doing so consists in an easier realisation of the method.

## 2. Materials and Methods

In this section, in a completely general setting, we derive a new upscaling framework for the estimation of key quantities and statistical properties of a system, be it natural or artificial. In particular, depending on the considered dataset, we will refer with the term items to the forest trees, the sent emails, the Twitter posts, the Wikipedia/Gutenberg words and the human polymorphisms. We then call type the label to which each item is associated: the species to which the tree belongs, the user who sent the email, the hashtag contained in the Twitter post, the word occurring in the Wikipedia pages or in the Gutenberg books and the nucleotide variant observed along a human genome.

### Theoretical Framework

Let us now assume we have access to a database containing a huge number of items, *N*, among which *T* types are observed. Thanks to this information, one may have insights into relevant statistical patterns characterising the global system, as the type frequency of frequencies distribution (TFFD), which describes how the total number *N* of items characterising the system are distributed among the *T* types. Our goal is to reduce the amount of information we need to store without preventing the possibility of an accurate statistical description of the whole database, in order not to miss key properties of the system under study. Let us thus extract from our original dataset *S* non-overlapping and equally-sized samples, each consisting of a random fraction *q* of the *N* system items. Moreover, let us suppose that, for each sample, we only save information on the list of distinct types observed among its qN items. Let us denote with $T^{\left(s\right)}$ the total number of different types found in the $s^{th}$ sample. We remark that, with this operation, we passed from the storage of *N* elements to the storage of $T^{\left(1\right)}+T^{\left(2\right)}+\cdots +T^{\left(S\right)}\ll N$ elements, apparently losing information on the total number of types *T* originally present in the database and on their frequencies. We wish to show that such fundamental statistics can be successfully recovered from the small amount of data we chose to store.

First, since we have preserved information on the type labels observed in each sample, it is possible to compute, at least locally, how the number of types grows with the fraction of the collected system items. More precisely, at each scale mq, m=1,2,⋯,S, one can compute the average number of types observed when aggregating *m* of the *S* samples. By denoting this latter quantity by $T_{mq}$, this procedure leads to the curve of points $\left(mq,T_{mq}\right)$ describing how the number of observed types increases with the portion of collected items. This curve is a well-studied pattern in theoretical ecology going under the name of species-accumulation curve or species-discovery curve [25,26,33,34]. In our more general setting, we will call it type-discovery curve (TDC). Our first goal was to find an estimator for *T*, the total number of types characterising the global system.

Following the statistical framework developed in [25,26,35], we started by considering the global TFFD, which is modelled according to a truncated negative binomial of parameters *r* and ξ:

(1) P(n|r,ξ)=c(r,ξ)n+r−1nξn(1−ξ)r,

where $c\left(r,\xi \right)={\left(1-{\left(1-\xi \right)}^{r}\right)}^{-1}$ is the normalisation constant. We recall that the *r* parameter, usually referred as the clustering coefficient in ecology due to its relation with individuals’ aggregation, lies in (0,+∞), whereas the ξ parameter stands in (0,1).

TFFD represents another widely-known pattern in ecology, the relative species abundance distribution, describing how the total number of trees populating an ecological system are distributed among the present species [25,26,36,37]. Distribution (1) has been proven to provide a very good description of the empirical data both within an ecological context and in human activity data [25,26,35]. Indeed, by tuning the value of the clustering parameter *r*, it allows us to capture both exponential (positive *r*) and heavy-tailed (r∈(−1,0)) behaviours. Moreover, it has the advantage of satisfying the so-called form-invariance property, which guarantees that it maintains the same functional form at different scales. Indeed, denoting with *p* a fraction of items randomly collected with respect to the total, the local TFFD is also a negative binomial with the same clustering parameter *r* as for the global scale and with a re-scaled $\xi_{p}$ parameter, which is a function of the global ξ and *p* [25,26,35]:

(2) $$\xi_{p}=\frac{\xi p}{1-\xi (1-p)}.$$

This form-invariance property has been empirically observed in data from both forest ecosystems [25,35] and human activities [35] and it made distribution (1) suitable for inference problems, since it allows us to find an easy estimator for the number of system types on the basis of a rigorous mathematical framework.

Indeed, let us now assume that one has collected $N_{p^{*}}$ items, together with the associated types. Then, one could perform a maximum-likelihood estimation to recover the *r* and ξ parameters via the fitting of the empirical TFFD, as done in [25,35]. Of course, this step could only be performed when local information on types abundances were available. In contrast, when only information on the presence/absence of types in samples is stored, an alternative approach is needed since the TFFD cannot be extracted from the data. In [26] a different method based on the fit of the local empirical TDC was been proposed to overcome this lack of information. Here we wished to combine the two approaches and to develop a novel estimator for *T*.

We started by computing the log-likelihood function associated to the TFFD (1), given the vector of abundances $\bar{n}=\left(n_{1},\;n_{2},\cdots ,\;n_{T}\right)$ of the *T* types occurring in the whole system. Denoting with $T_{n}$ the number of types having abundance *n* at the global scale, the log-likelihood function associated to (1) can be computed as follows:

(3) Lr,ξ(n¯)=∏t=1TP(nt|r,ξ)==∏nP(n|r,ξ)Tn==Tlogc(r,ξ)+rTlog(1−ξ)++Nlogξ+∑nTnlogn+r−1n.

The maximum-likelihood estimators for the TFFD parameters ξ and *r* are those maximizing the log-likelihood function:

(4) $$\begin{array}{cc} r & =\underset{r\in (-1,\infty )}{arg max}L_{r,\xi }\left(\bar{n}\right)\\ \xi & =\underset{\xi \in (0,1)}{arg max}L_{r,\xi }\left(\bar{n}\right). \end{array}$$

In particular, since both *r* and ξ are continuous parameters, the maxima of $L_{r,\xi }\left(\bar{n}\right)$ are critical points and can thus be found by setting the partial derivative of $L_{r,\xi }\left(\bar{n}\right)$ equal to zero. Focusing on $\frac{\partial }{\partial \xi }L_{r,\xi }\left(\bar{n}\right)$, one ends up with the following equation:

(5) $$\begin{array}{c} -Tr\frac{{\left(1-\xi \right)}^{r-1}}{1-{\left(1-\xi \right)}^{r}}-\frac{Tr}{1-\xi }+\frac{N}{\xi }=0. \end{array}$$

Under the assumption of N≫0, Equation (5) can be understood in a deterministic sense. Indeed, since the TFFD expressed in (1) is uni-modal, the relative errors on the maximum-likelihood estimators of the parameters are small [38]. Equation (5) can thus be inverted to heuristically obtain an estimator for *T*, hereby denoted with $\hat{T}$:

(6) $$\hat{T}=N\frac{(1-{\left(1-\xi \right)}^{r})\left(1-\xi \right)}{\xi r}.$$

Let us see how to exploit the above formula. We have already noticed that having stored information on the presence/absence of types in *S* scattered samples of a system each containing a fraction *q* of the global number of the system items is sufficient to construct the empirical TDC from the local scale *q* up to the scale $p^{*}:=Sq$. Moreover, with similar arguments, such as those used to obtain Equation (6), we have that, at any given sub-scale p=mq, m=1,⋯,S the number of items $T_{p}$ at that scale is given by

(7) $${\hat{T}}_{p}=N_{p}\frac{\left(1-{\left(1-\xi_{p}\right)}^{r}\right)\left(1-\xi_{p}\right)}{\xi_{p}r},$$

where $N_{p}$ is the total number of items observed at the scale *p*. Such a quantity can also be expressed as a function of the total number of items counted by aggregating all the *S* samples. In fact, let us call $N^{*}$ such a quantity, which corresponds to the maximum available TDC scale, $p^{*}$. Then we have the relation

(8) $$N_{p}=pN=p\frac{N^{*}}{p^{*}},$$

which, inserted into (7), leads to

(9) $${\hat{T}}_{p}=\frac{pN^{*}}{p^{*}}\frac{\left(1-{\left(1-\xi_{p}\right)}^{r}\right)\left(1-\xi_{p}\right)}{\xi_{p}r},$$

Finally, plugging Equation (2) into Equation (9), we get

(10) $${\hat{T}}_{p}=\frac{N^{*}}{p^{*}}\frac{\left(1-\xi \right)}{r\xi }\left[1-{\left(\frac{1-\xi }{1-\xi (1-p)}\right)}^{r}\right].$$

Thus, we can fit the empirical TDC up to the scale $p^{*}$ through Equation (10) above to obtain an estimate $\hat{r}$ and $\hat{\xi }$ of the TFFD parameters at the global scale. At this point, we have all the ingredients to upscale the empirical TDC up to the global scale. In particular, to recover the total number of types *T* of the system, it suffices to insert the fitted parameters into Equation (6). Moreover, let us observe that, although we only saved information on the presence/absence of types in samples, since $\hat{r}$ and $\hat{\xi }$ are the negative binomial TFFD parameters, we can also retrieve the distribution of items among the estimated $\hat{T}$ types.

In the following section. we will show how estimator (6) performed against the two datasets from tropical rainforests and the six from social activities.

## 3. Results

### 3.1. Tests on Rainforest and Human-Activity Data

In order to test the performance of estimator (6), we first applied it to six datasets, ranging from forest ecosystems to human activities (see Table 1).

We considered a random fraction $p^{*}=10\%$ for the ecological data and a random fraction $p^{*}=5\%$ for the social data, miming the data reduction process. We then sub-sampled each limited dataset at 100 equally-spaced scales *x* ranging from 0.01 to 1, each corresponding to sampling a fraction $p=xp^{*}$ of the global number of items. We thus constructed the empirical TDC of each system up to the $p^{*}=10\%$ or $p^{*}=5\%$ scale, counting how many different types are found at each sub-scale *p* and we fitted it via Equation (10). We thus got the values of $\hat{r}$ and $\hat{\xi }$ that best described the observed pattern. We finally exploited our estimator (6) to recover the total number of types catalogued within each system and we compared the results with the actual known values. We repeated the procedure ten times for each of the six datasets. In Table 1 we reported the average values among the ten replicates of the fitted parameters and the estimates of the total number of types together with the relative percentage errors with respect to the true values. As we can see, the new estimator performs quite well for retrieving the total number of types within each considered system from the storage of a limited portion of it.

We compared the obtained results with those obtained in [26] for the forest ecosystems and in [35] for the human-activity data, respectively (see Table 2 and Discussions), where other upscaling approaches based on the negative binomial model have been proposed. We found that, although the information used as input was further reduced with respect to the previous methods, the novel method gave comparable results for all datasets, except for the BCI, against which it performed worse than the others.

**Figure 1:**
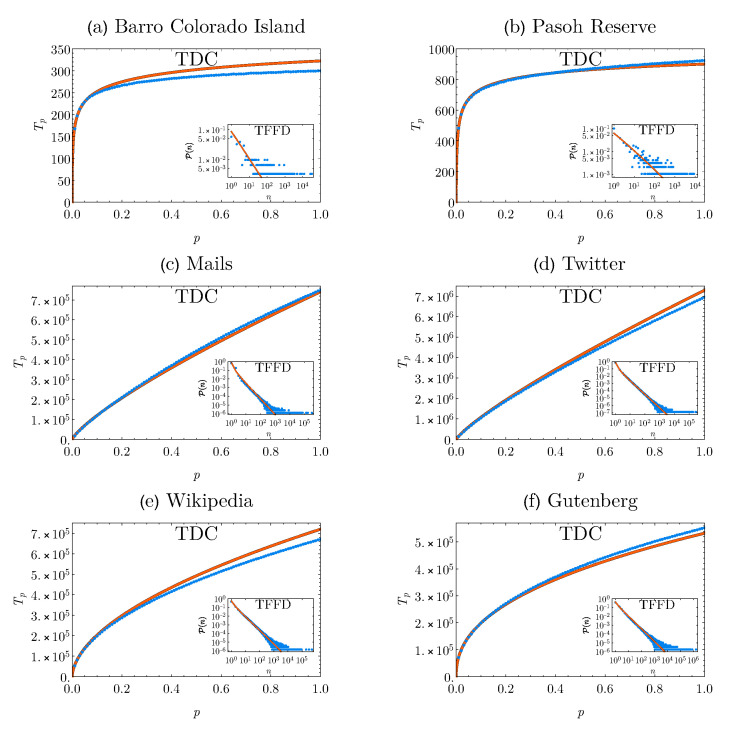
**Empirical vs predicted patterns.** Plots of the empirical TDC (blue dots) and the predicted ones (solid orange line) for the datasets of the two tropical rainforests (panels (**a**,**b**)) and of the four human activities (panels (**c**–**f**)). In the insets, the empirical TFFDs (blue dots) and the predicted ones (solid orange line) are displayed. Both for the TDC and the TFFD, the predicted curves are computed as the average of the single TDCs and TFFDs obtained from ten independent replications of the procedure. In particular, the theoretical estimates of the TFFD patterns are obtained by evaluating Equation (1) with the parameters coming from the fit of the local TDCs displayed in Table 1.

Figure 1 shows the empirical TDC and TFFD patterns for the entire datasets against those we reconstructed by inserting the fitted parameters into (10) and (1), respectively. At each scale for the TDC and at each abundance for the TFFD, the corresponding predicted points displayed represent the averages among the ten replicates. We can see from Figure 1 that, although the functional form of the TDC given by the estimator is independent of the origin of the data we aim to describe, the empirical patterns display a different trend between the ecological and human-generated datasets. This is reflected by different values of the clustering parameter $\hat{r}$ in the TFFD pattern our estimator retrieves. In fact, it acquires positive values for the rainforests cases and negative values in the four human activities networks considered (see Table 1).

We also wish to stress the fact that the theoretical predictions for both the patterns (corresponding to the solid lines in Figure 1) are not the result of the fit of the global empirical TDCs, but they are computed by fitting the local ones up to the smaller sample scales $p^{*}$ (equal to 10% for the forests and to 5% for the human datasets). Nevertheless, the theoretical predictions and empirical observations are in good agreement, both for the TDC and TFFD patterns. We finally remark that, as we can see from the inset plots in Figure 1, our procedure is not only able to accurately reproduce the TDC behaviour, but it also faithfully retrieves the TFFD pattern, thus allowing us to recover abundant information from the storage of binary presence/absence data.

### 3.2. Application to Human Single Nucleotide Polymorphism Data

Human single nucleotide polymorphisms (SNP) are substitutions of a single nucleotide along the genome sequence that may occur in both non-coding and coding DNA regions, potentially leading, in this latter case, to erroneous protein productions. A given SNP is characterized by the position at which it occurs as well as by the specific nucleotide mutation it brings with respect to the reference genome [39]. With an average occurrence frequency of one every 1000 nucleotides, SNPs constitute the most abundant sources of genetic variation among people and they have become particularly relevant in genome association studies which aim at identifying high-resolution markers related to complex diseases via gene causal mapping [40,41]. Therefore, predicting the existence of unseen SNPs and gaining an estimate of their abundance within a genetic region may be informative in these kinds of analyses and may help to highlight phenotypic variation and to detect genetic patterns for treatment responses [42,43]. Due to their importance in medical studies, genomic data have been recently collected at an extraordinary high rate, also thanks to the modern sequencing techniques nowadays available to the scientific communities. This has resulted in huge databases needing non-trivial resources to be investigated. Here, inspired by the results obtained in the ecological and social contexts, we wish to apply our upscaling procedure in this context of genomic variants to both address the problem of data storage and to open the possibility of new insights into this kind of databases. In particular, we analysed a SNP dataset from the 1000 Genome Project [29,30,31]. The project, carried out from 2008 to 2015, had the scope of creating the largest public catalogue of genetic variation in human population. Indeed, the revolution accomplished in genomic analyses by the development of modern high-throughput sequencing techniques allowed to sequence genomes from a huge number of individuals, paving thus the way for the creation of a comprehensive resource on human genetic variation.

The dataset analysed here contained 58,681 SNPs observed between positions 2,655,180 to 28,770,931 of the *Y* chromosome of 1233 sequenced males. Given the fact that this chromosome is present with a single copy in male individuals, in each subject any SPN may be either observed once or not at all. In order to test estimator (6), we proceeded as follows. We firstly extracted a random fraction $p^{*}=5\%$ of the total number of nucleotide variations *N* = 839,459 observed within the 1233 subjects. We then constructed the local TDC at 100 equally-spaced scales from $0.01p^{*}$ to $p^{*}$, similar to what we did for the natural ecosystems and the human-activity databases. We then fitted the resulting pattern via Equation (10) in order to obtain the values of the local TFFD parameters $\hat{r}$ and $\hat{\xi }$. Finally, we applied (6) to recover of the total number of items *T* (number of different SNPs present in the database) at the global scale—i.e., of the original big dataset. As for the other analysed systems, we repeated the procedure ten times. In Table 3, we reported the average values among the ten replicates of the fitted parameters and the estimates for *T* together with the relative percentage errors with respect to the true values. We conducted the test also for other values of the local scale $p^{*}$. In particular, in Table 3, we inserted the results for a storaged fraction $p^{*}=20\%$, whereas in Table 4 we displayed the recovered values for *T* and the corresponding relative errors obtained at different sub-scales ranging from $p^{*}=5\%$ to 50%. We can notice that, as it would be desirable for an upscaling estimator, which explicitly depends on the stored data fraction, the errors for the estimate of the number of types decreases with the sub-scale $p^{*}$ considered, since the more information we store, the better the recovering procedure perform should be.

**Figure 2:**
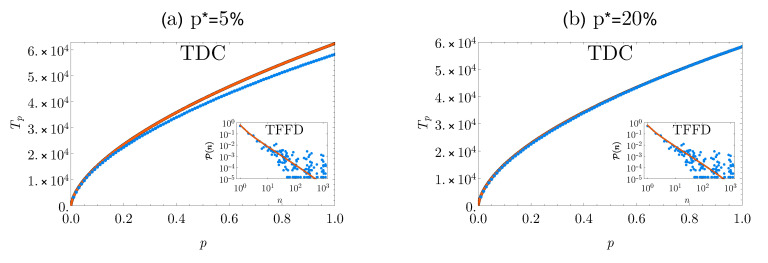
**Empirical vs. predicted patterns for the SNP dataset.** Plots of the empirical TDCs (blue dots) and the predicted ones (solid orange line) for the SNP data at two different sampling scales $p^{*}=5\%$ (panel (**a**)) and $p^{*}=20\%$ (panel (**b**)). In the insets, the empirical TFFDs (blue dots) and the predicted ones (solid orange line) are displayed. Both for the TDC and the TFFD, the predicted curves are computed as the average of the single TDCs and TFFDs obtained from ten independent replications of the procedure.

In Figure 2, we plotted the empirical TDC and TFFD patterns for the entire datasets against those retrieved from the scales $p^{*}=5\%$ and $p^{*}=20\%$. As for the other datasets, the points displayed represent the averages values of $\hat{T_{p}}$ among the ten replicates.

**Figure 3:**
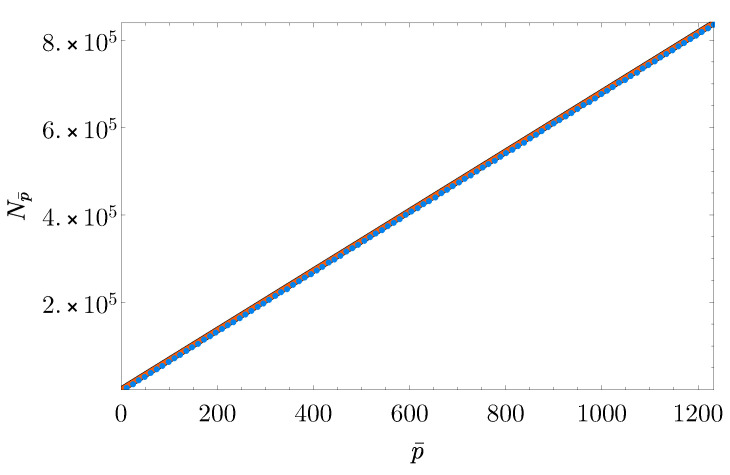
**Linear fit between the number of study subjects and observed variants.** Blue points correspond to pairs ($\bar{p}$, $N_{\bar{p}}$), where $\bar{p}=1,\cdots ,1233$ is the number of sequenced individuals and $N_{\bar{p}}$ is the average number of observed mutations over 100 different $\bar{p}$ samples of randomly chosen subjects. The orange line is the fitting line obtained from linear regression (slope = 680.8, $R^{2}\approx 1$).

We remark that, up to now, we considered as a sample at scale $p^{*}$ the stored portion of the total number of variants observed among the sequenced population. Therefore, the TDC patterns shown in Figure 2 represent how $T_{p}$ increases with the fraction *p* of *N*, the total number of variations observed. However, in real applications, it would be more intuitive and useful to recover information on (and eventually predict at even larger scales) how the number of different genomic variants grows as a function of the number of sequenced genomes—i.e., with the number of participants to the study, *I*. Thus, let us define $\bar{p}$ as the number of sequenced individuals corresponding to a fraction *p* of *I*: $\bar{p}=pI$ with $\bar{p}=1,\cdots ,I=1233$. Let $N_{\bar{p}}$ be the corresponding total number of variants observed among their genomes. The empirical values for $N_{\bar{p}}$ at different $\bar{p}$ were computed as follows. Given $\bar{p}$, we sampled 100 different random combinations of $\bar{p}$ subjects among the total ones and computed, for each subset, the total number of observed mutations among its $\bar{p}$ individuals. Finally we set $N_{\bar{p}}$ as the average of this quantity among the 100 samples. We found that $N_{\bar{p}}$ strongly correlates to $\bar{p}$ via a linear relation. In particular, performing a linear fit between the two variables and constraining the intercept to equal zero, we found a slope of 680.8 (*p*-value $<10^{-16}$) with a corresponding R-squared value $R^{2}\approx 1$ (see Figure 3).

**Figure 4:**
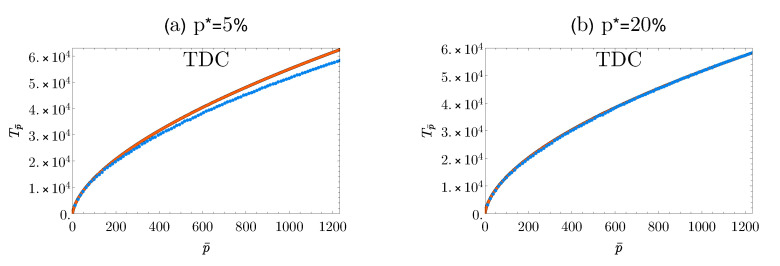
**Empirical vs. predicted TDC patterns as a function of the number of study subjects.** Plots of the empirical TDCs (blue dots) and the predicted ones (solid orange line) for the SNP data at two different sampling scales $p^{*}=5\%$ (panel (**a**)) and $p^{*}=20\%$ (panel (**b**)). The predicted curves are computed from those displayed in Figure 2 by exploiting the linear relation between the number of sequenced genomes $\bar{p}$ and the average number of variants observed within them, $N_{\bar{p}}$, shown in Figure 3.

Note that this result allows us to estimate how the number of different mutations (here denoted with $T_{\bar{p}}$) grows with the number of sequenced individual genomes, $\bar{p}.$ Indeed, thanks to the linear relation between $N_{\bar{p}}$ and $\bar{p}$, we can easily connect the total number of mutations *N* with the total number of study subjects *I* via N=680.8I. Thus we have that the percentage of mutations, *p*, can be rewritten via the following: $p=\bar{p}/I=680.8\bar{p}/N$. This relation allows us to write the TDC in (10) as a function of $\bar{p}$ by just re-scaling the independent variable. In Figure 4 we compared the pattern recovered with this procedure against the empirical points, computed as the number of different mutations observed among a sub-sample of dimension $\bar{p}$, averaged over 100 independent sampling procedures of the $\bar{p}$ subjects. As we can see, our prediction faithfully reproduces the empirical data. We remark that here we only tested the performance of the framework in recovering statistical patterns of interest of big databases from the storage of a limited portion of them. Nevertheless, one could, of course, apply the very same approach with the aim of upscaling such patterns from the data scale up to any desired scale, thus extending the range of applicability of the method so to infer the statistical properties of an entire population, beyond the scale at which current technologies allow us to collect data.

## 4. Discussion

In this study, we adopted an upscaling perspective from ecology to face the problem of retrieving global statistical properties of big data from a reduced amount of information. Within this scope, we proposed a new estimator able to retrieve both the number of a system’s types and the related patterns derived from the same statistical framework of [25,26,35]. More precisely, starting from a negative binomial model for the type frequency of frequencies distribution and inverting the maximum-likelihood equation for the ξ parameter, we obtained an analytic relation between the number of types at different scales and the global TFFD parameters. This result allows for the recover of the total number of types in a system, be it a natural ecosystem or a social network, when the only stored information is in the presence/absence of types in small local samples. Moreover, it also permits us to retrieve information on different patterns of interest, as the type–discovery curve and the type frequency of frequencies distribution up to any desired scale, allowing us thus to have insights also on the abundance distribution of items among types, important information which was lost with the reduction processing of the original data. This makes the approach similar to what was proposed in [26] for ecological systems, where the form-invariance property of the negative binomial distribution was exploited to derive an analytic expression of the species–accumulation curve. However, such form has an explicit dependence on the total number of species, *T*, which is, of course, unknown. To overcome this problem, it is necessary to re-scale all local sub-scales *p* so to refer to $p^{*}$ as the global scale. One can then fit the empirical type-discovery curve to obtain an estimate for the parameters *r* and $\xi_{p^{*}}$ that best describe it and later to infer the global ξ by inverting Equation (2). Here, instead, the discovery curve comes to depend only on the total number of items, *N*, present in the global system, information which is more often accessible. No re-scaling is therefore needed, so that the fitting of the type–discovery curve directly allows for the recover of the global frequency of frequencies distribution parameters.

As for the social activity systems, the approach adopted here relies on different starting information with respect to those assumed in [35]. Indeed, in [35], the two parameters were obtained via the fitting of the local type frequency of frequencies distribution, for which abundance information are therefore essential. Here, instead, we fit the empirical discovery curve, so that binary information on the presence/absence of species is all that is needed to store.

By comparing our results with those obtained in [26] for the rainforests data we found that (see also Table 2), although the relative error between the true number of types at $p^{*}$ and the one predicted are comparable for the Pasoh forest (equal to 1.8% for the method in [26] against 2.9% here), for the BCI the new estimator leads to an error of −7.1% against the 1.8% found in [26]. This result is in agreement with the estimates of the TFFD parameters. Indeed, for the Pasoh forests, the values of both *r* and ξ are similar between the two methods, (r=0.24, ξ=0.9998 in [26] against r=0.20ξ=0.9992 found here), whereas for the BCI dataset, the clustering parameter obtained here resulted one order of magnitude lower than the one fitted in [26] (r=0.20, ξ=0.9991 in [26] against r=0.073 and ξ=0.9998 here). As for the human activity data, we benchmarked our estimator with the one adopted in [35]. Remarkably, although our framework reduces the needed amount of input information to binary presence/absence data, for all datasets both the values of *r* and ξ and the corresponding errors between predicted and true values of *T* between the two approaches referenced in [35]. Only for the emails dataset, the new error, although small, is ten times the previous one (1.5% against 0.11%). We then applied our procedure within the new context of genomic DNA. More precisely, we considered data from the 1000 Genome Project containing the single nucleotide polymorphisms along the *Y* chromosome of more than a thousand subjects. We found that, also in this case, the negative binomial model allows for the recovery of both the number of types and their relative patterns (type–discovery curve and type frequency of frequencies distribution) after the data reduction process. We remark that the application of our method in genomics to recover SNPs from smaller databases as well as to possibly predict the unseen SNPs beyond the up-to-now collected data scale follows a current trend in clinical research. Indeed, nowadays many efforts are put in gaining accurate assessment and quantification of DNA mutation variability and, more specifically, of intra-tumour heterogeneity, due to their clinical implications [44]. For instance, the level of genetic diversity within an individual tumour could be informative of aspects of internal dynamics, dealing with future evolution and treatment responses, that should be taken into account in ad hoc therapeutic designing [43,45]. However, this latter is usually based on results collected from single biopsy samples, that are statistically unlikely to accurately capture the complete mutational profile of the cancer. Thus, there is an increasing need for technologies capable of obtaining a more reliable and extended genetic description of the disease that may help in taking crucial medical decision, with no adding cost, time waste and ethic implications as drawbacks. Our method, specifically developed for recovering global measurements from local ones, could meet the mentioned real need and could prove to be a useful tool within such a clinical context. Finally, we also explored how our procedure performed at different data storing fractions $p^{*}$. As expected, we observed that, by increasing the portion of the dataset used as input, the framework performs better. Therefore, depending on the level of precision one would like to reach in the final estimates and the amount of storage space and computational resources available, one must tune the quantity of data s/he needs to collect and save. In other words, the accuracy of the global description of the system under study and the reduction process of the corresponding data are in trade-off. As an example, for the single nucleotide polymorphisms dataset considered heref we found a relative error of only 0.3% when storing 50% of the original database, against an error of 6.4% when decreasing, by ten times, the input data fraction.

## 5. Conclusions

Big data revolution had a major impact on all scientific research fields. One of the most important issue emerged from it stands on the fact that huge amounts of data require lots of space capacity to be stored and advanced techniques to be analysed. Sometimes it is necessary to reduce the quantity of available data in order to be able to manage them. This process, of course, may lead to the loss of details. Therefore, it is essential to design novel methods allowing for the recovery, with small errors, of all the information contained within the original big dataset only having information on a smaller sample of it.

Here we exploit the ecological idea of upscaling to deal with the big data storage and analysing problems in effective way. More precisely, we propose a novel procedure based on a maximum-likelihood-inspired estimator for the number of types in a system, which, starting from reduced binary information in the presence/absence of types across small scales, is able to recover global information of interest in the original dataset.

We tested our method on several big datasets from different research fields. In particular, we mimic a reduction in the storage for the datastream by considering only small random fractions of the originally collected data and try to retrieve global information on the entire dataset. In all the cases we studied, we found that our procedure allows for the successful recovery, with acceptable errors, of some major statistical patterns of the original system, as the type frequency of frequencies distribution and the discovery curves.

Thanks to the generality of the setting, within which our estimator is derived, our approach could be of help whenever the massive quantity of data, nowadays available, comes at a too high cost in terms of storage and energies needed to manage and analyse them. Indeed, our method allows for the reduction in big databases without the loss of essential information, which could be crucial to have a clearer view of the systems they represent. These results pave the way for the application of our approach in contexts beyond those of ecology and human behaviour, for which it was originally conceived.

## Figures and Tables

**Table 1:** **Upscaling results for the rainforest and human-activity datasets.** For each dataset, the number of different types and items, *T* and *N*, respectively, are reported together with the average of the TFFD parameters, $\hat{r}$ and $\hat{\xi }$, at the global scale. These latter were obtained by fitting the local TDCs constructed from the ten random $p^{*}$ samples of each dataset trough Equation (10). Note that the $\hat{r}$ parameter was positive for the two forests and negative for the human-activity datasets, consistently with the results presented in [35]. Moreover, in all of the six datasets $\hat{\xi }$ is very close to 1. Finally, we inserted the average predictions $\hat{T}$ of the number of different types at the global scale using estimator (6) and their relative percentage errors with respect to the real values, *T*.

Dataset	T	N	$\hat{r}$	$\hat{\xi }$	$\hat{T}$	Relative Error
BCI	301	222,602	0.073 ± 0.002	$0.999769\pm 10^{-6}$	322.4±0.6	(−7.1±0.2)%
Pasoh	927	310,520	$0.2022\pm 6\cdot 10^{-4}$	$0.999235\pm 10^{-6}$	900.4±0.4	(2.87±0.04)%
Emails	752,299	6,914,872	$-0.7899\pm 8\cdot 10^{-4}$	$0.9999921\pm 2\cdot 10^{-7}$	741,036 ± 3646	(1.5±0.5)%
Twitter	6,972,453	34,696,973	$-0.8275\pm 2\cdot 10^{-4}$	$0.9999605\pm 3\cdot 10^{-7}$	729,288 ± 8357	(−4.6±0.1)%
Wikipedia	673,872	29,606,116	$-0.545\pm 10^{-3}$	$0.9999241\pm 7\cdot 10^{-7}$	720,432 ± 3295	(−6.9±0.5)%
Gutenberg	554,193	126,289,661	$-0.4203\pm 8\cdot 10^{-4}$	$0.9999817\pm 10^{-7}$	532,555 ± 2110	(3.9±0.4)%

**Table 2:** **Comparative results for rainforest and human-activity datasets.** Here we show the predictions for the Negative Binomial parameters, $\hat{r}$ and $\hat{\xi }$, and the relative error of the estimates $\hat{T}$ with respect to the true value *T*. In particular, we compared the results obtained by using the estimator Equation (6) with some benchmark results provided by Refs. [26,35] for the rainforest and human-activity datasets, respectively.

Dataset	Estimator	$\hat{r}$	$\hat{\xi }$	Relative Error
BCI	Equation (6)	0.073	0.9998	−7.1%
	Ref. [26]	0.20	0.9991	1.8%
Pasoh	Equation (6)	0.20	0.9992	2.9%
	Ref. [26]	0.24	0.9998	1.8%
Emails	Equation (6)	−0.795	∼1	1.5%
	Ref. [35]	−0.798	∼1	0.11%
Twitter	Equation (6)	−0.824	∼1	−4.6%
	Ref. [35]	−0.828	∼1	3.33%
Wikipedia	Equation (6)	−0.543	∼ 1	−6.9%
	Ref. [35]	−0.544	∼1	6.11%
Gutenberg	Equation (6)	−0.426	∼1	3.9%
	Ref. [35]	−0.420	∼1	−2.3%

**Table 3:** **Upscaling results for the SNP datasets.** For different percentages $p^{*}$ of the sampled variants, we reported the total number of items present in the gloabal database, *T*, the number of different types, *N*, the average of the TFFD parameters $\hat{r}$ and $\hat{\xi }$ obtained by the fit of the local TDC curves and the average predictions $\hat{T}$ of the number of different types at the global scale via (6), together with the corresponding relative percentage errors with respect to the real values, *T*.

Fraction p*	T	N	$\hat{r}$	$\hat{\xi }$	$\hat{T}$	Relative Error
5%	58,671	839,459	−0.597±0.003	$0.99955\pm 10^{-5}$	62,414±816	(−6.4±1.4)%
20%	58,671	839,459	−0.569±0.002	$0.99942\pm 10^{-5}$	58,297±370	(0.6±0.6)%

**Table 4:** Sensitivity on $p^{*}$ for the SNP dataset. We reported the value of the total number of types at the global scale predicted by our method, $\hat{T}$, together with the corresponding relative percentage errors with respect to the real values, *T*, for different choices in the sampling percentage $p^{*}$.

$p^{*}$	$\hat{T}$	Relative Error
5%	62,414 ± 816	(−6.4±1.4)%
10%	60,471 ± 539	(−3.1±0.9)%
20%	58,297 ± 370	(0.6±0.6)%
30%	58,070 ± 197	(1.0±0.3)%
40%	58,371 ± 143	(0.5±0.2)%
50%	58,504 ± 111	(0.3±0.2)%

## Supplementary Materials

All data used in this study are available online or upon request. In particular, the Pasoh and Barro Colorado Island datasets were provided by the Center of Tropical Research Science of the Smithsonian Tropical Research Institute (https://stri.si.edu/). The email dataset can be found in [27], while the other human activity data (Twitter, Wikipedia and Gutenberg) are publicly available [28]. Finally, data on human polymorphisms can be freely downloaded from the 1000 Genome Project website (see ftp://ftp.1000genomes.ebi.ac.uk/vol1/ftp/release/20130502).

## Abbreviations

The following abbreviations are used in this manuscript:

TFFD	Type Frequency of Frequencies Distribution
TDC	Type-Discovery Curve
SNP	Single Nucleotide Polymorphism
